# Changes in the functional traits of *Thymus mongolicus* along environmental gradients and factors influencing the traits of Northern China

**DOI:** 10.3389/fpls.2025.1596849

**Published:** 2025-05-20

**Authors:** Wentao Mi, Hao Zheng, Haixuan Zhang, Wanyu Zhang, Yuan Chi, Weibo Ren

**Affiliations:** Inner Mongolia Key Laboratory of Grassland Ecology and the Candidate State Laboratory of Ministry of Science and Technology, School of Ecology and Environment, Inner Mongolia University, Hohhot, China

**Keywords:** adaptability, functional traits, *Thymus mongolicus*, environmental gradient, leaf

## Abstract

*Thymus mongolicus* is a common medicinal and edible plant in grasslands of northern China. However, previous studies were limited to the production and utilization value of *T. mongolicus*, and only few studies have paid attention to the changes in the functional traits of wild *T. mongolicus* on a large scale. Therefore, this study examined 145 *T. mongolicus* specimens collected from northern China. By measuring 10 functional traits, including leaf length, leaf width, and leaf area, we analyzed the interplay between functional traits as a response to changes in environmental factors. Significant differences were observed between the eastern and western populations of *T. mongolicus*. Functional traits exhibited pronounced latitudinal and longitudinal gradients. With increasing latitude and longitude, *T. mongolicus* tended to change its functional traits to adapt to environmental changes. In high-temperature and high-rainfall environments, *T. mongolicus* developed larger leaf areas and longer leaves. Random forest analysis identified temperature within the latitudinal and longitudinal context as the primary driver of functional trait variation. Furthermore, interdependencies among functional traits were evident, with specific traits—such as stem length, leaf width, and leaf area—emerging as central to the adaptive process. These findings elucidate the mechanisms and key factors underlying the functional trait adaptation of *T. mongolicus*, providing critical insights for the breeding of region-specific varieties in China.

## 1 Introduction

Functional traits are the most intuitive way to study the response of plants to environmental changes and have always been an important research direction in ecology (Cornelissen et al., 2003; Reich et al., 2003). Over the past two decades, research on functional traits has spanned multiple levels—ranging from physiological to organ-level processes, from individual plants to ecosystems, and from aboveground to belowground interactions—extending into diverse ecological disciplines (Sobral, 2021). The main research areas are as follows: (1) the internal relationships and trade-offs among functional traits (Jin et al., 2024; Zhang et al., 2024); (2) the distribution patterns and variation in traits along environmental gradients (Hulshof et al., 2013; Wieczynski et al., 2019); (3) responses of traits to disturbances such as drought, fire, and grazing (Kerr et al., 2023; Wang et al., 2023); (4) studies on functional diversity (Díaz and Cabido, 2001); and (5) the mechanisms underlying species coexistence (Mello et al., 2020). The leaf traits of woody plants are closely related to changes in environmental factors such as light and precipitation. In temperate regions, woody plants usually have larger leaves and a higher specific leaf area (LA) to improve the efficiency of photosynthesis in a longer growing season. In arid or low-light environments, these plants tend to exhibit smaller leaves and a lower specific LA to reduce water evaporation and enhance drought resistance. The regulation of this trait enables woody plants to effectively respond to environmental changes and maintain survival and stability under diverse ecological conditions (Vilà et al., 2015). In addition to woody plants, bryophytes and herbaceous plants also showed similar regular changes (Candeias and Fraterrigo, 2020; Souza et al., 2020).

Leaves represent the most evolutionarily plastic organ in plants, playing vital roles in photosynthesis, respiration, and evapotranspiration—key processes for maintaining plant life (Koyama et al., 2012). Leaf size reflects a plant’s light-capture efficiency, carbon acquisition capacity, and environmental adaptations, revealing a trade-off between carbon assimilation and water use (Onoda et al., 2012). This trade-off is central to regulating leaf temperature and photosynthetic efficiency under varying climatic conditions, forming a cornerstone of plant ecological strategies. Leaf morphological characteristics serve as intuitive indicators of plant adaptability to specific habitats (Henn et al., 2018). The dynamic changes in leaf shape reflect how plants respond to environmental challenges, showcasing fundamental ecological behaviors and strategies (Chitwood and Sinha, 2016; Fritz et al., 2018). For instance, the same plant can produce different leaf shapes across distinct environments, with each shape tailored to specific adaptive functions (Li et al., 2019). Stem is the main transport network for carrying leaves. The length of the stem may directly determine the number of leaves, LA, and other functional traits. As a “bridge” connecting both aboveground leaves and underground roots, the stem plays a key role in plant nutrient transport (Huang et al., 2016; Miao et al., 2023; Sun et al., 2019). Additionally, because the stem of thyme can grow adventitious roots and expand the living area of self-growth, the length of the stem reflects the expansion ability of *T. mongolicus*.

*Thymus mongolicus* (Ronniger) Ronniger, a member of the Lamiaceae family, is a perennial small semi-shrub. Its slender, woody stems bear opposite leaves that are small, narrow, and oval-shaped, with surfaces covered in small glands that secrete essential oils with a strong aroma. The flowers of *T. mongolicus* are small, dense, and light purple or pink, with occasional white variants. These flowers bloom in spring or summer, featuring a long flowering period and significant ornamental value (Sun et al., 2023). The natural distribution of *T. mongolicus* Shanxi, Hebei, Inner Mongolia, and other regions in China (Li and Hedge, 1994). It is widely distributed in various habitats such as sandy land, grassland, understory and forest-grass transition zone. These regions and habitats have significant differences in environmental factors such as altitude, precipitation, and temperature. In the field sampling survey conducted in this study, the functional traits of *T. mongolicus* in different regions showed significant differences. However, the mechanism underlying the changes in functional traits needs further exploration. The functional traits of plants are primarily influenced by light, temperature, and water, with different environmental conditions driving diverse adaptation strategies. The relationships between “trait-environment” and “trait-trait” interactions illustrate the principles of optimal adaptation for plant growth in natural conditions. However, previous studies have focused more on the medicinal and edible research of *T. mongolicus*, limited research has explored the large-scale adaptation of *T. mongolicus* functional traits to environmental factors or how these traits are internally balanced. This study collected *T. mongolicus* samples from 145 sites across northern China to address two key scientific questions: (1) How do the functional traits of *T. mongolicus* adapt to vary across heterogeneous environments? (2) How are *T. mongolicus* functional traits internally each other during heterogeneous environmental adaptation?

## 2 Materials and methods

### 2.1 *T. mongolicus* collection and transplanting

From 2022 to 2023, we collected *T. mongolicus* samples from 145 sampling sites across Inner Mongolia, Shanxi, Hebei, and Liaoning. The *T. mongolicus* at each sampling point was considered a material, and 8 individual plants were collected from each material. In this study, 145 sites were numbered one by one, according to the order of sampling, numbered from Thy1 to Thy145. The sampling sites were spaced at least 30 km apart to avoid spatial redundancy. To minimize duplication, samples were collected from individuals at least 5 m apart. For each site, we recorded latitude, longitude, altitude, habitat characteristics, and associated companion species. Because the field sampling lasted too long, the field measurement led to a large difference in the measurement time between different materials, resulting in differences in functional traits. To maintain a consistent measurement time and environment, the collected plants were placed in pots, watered, and then immediately transplanted to the Grassland Ecosystem Research Station at Inner Mongolia University (Xilinhot, coordinates 44°10′4″N, 116°28′56.8″E). All samples were maintained under uniform field management, with regular weeding but no fertilization, in preparation for subsequent uniform measurements. According to the location of the sampling points, we divided the sampling area into eastern and western regions, and there were significant differences between the east, west and center environmental factors (**Supplementary Table S1**).

### 2.2 Measurement of functional traits

In July 2024, we measured 10 functional traits for each plant in 145 samples (**Table 1**). To prevent experimental errors due to time-of-measurement variability, all samples were processed on the same day. Plant height was measured, and four branches from each plant—healthy, pest-free, and of uniform length—were selected for analysis. These branches were stored in water-sealed bags under refrigeration. Stem lengths were measured using an electronic vernier caliper. For leaf traits, we selected the three largest pairs of opposite leaves on each branch, ensuring they were healthy and pest-free. LA, leaf length, and leaf width (LW) were measured using ImageJ software, with the data being exported to Excel (Microsoft 2021) for further analysis. The remaining leaves on each stem were counted, along with the number and lengths of stem nodes, measured with a vernier caliper. Leaf shape factor was calculated using a defined formula: lSF=(4π*0.8WL)/(L+W)^2^, where L is leaf length (LL) and W is LW.

**Table 1:** Measured functional trait indicators, units, and abbreviations.

Traits	Unit	Abbreviation
Leaf number per branch	–	LN
Leaf area	mm^2^	LA
Leaf length	mm	LL
Leaf width	mm	LW
Leaf shape factor	–	LSF
Leaf length:leaf width	–	L:W
Height	cm	–
Stem length	mm	SL
Stem node number	–	SN
Stem node length	mm	SNL

### 2.3 Environmental factor extraction

Plant functional traits adapt to environmental conditions over the long term. Accordingly, we selected 10 stable environmental factors for predictive modeling (**Table 2**). These factors were categorized into spatial, topographic, and climatic variables. Longitude, latitude, and altitude were derived from GPS data, while the remaining factors were sourced from WorldClim (https://www.worldclim.org/).

**Table 2:** Factors and classifications included in the study.

Factors	Unit	Abbreviation	Classification
Longitude	°	–	Spatial
Latitude	°	–	Spatial
Elevation	m	–	Topographic
Slope	°	–	Topographic
Aspect	°	–	Topographic
Mean annual temperature	°C	MAT	Climate
Mean annual precipitation	mm	MAP	Climate
Solar radiation	kJ m^−2^ day^−1^	SR	Climate
Growing maximum temperature	°C	G.MaxT	Climate
Growing minimum temperature	°C	G.MinT	Climate

### 2.4 Data analysis

The data analysis in this study was conducted using the R programming language (R version 4.4.1; R Core Team, 2023). The mean (mean), standard deviation, and coefficient of variation (CV) were calculated with the *dplyr* package (Wickham et al., 2023). Principal component analysis (PCA) was performed using the *factoextra* package (Kassambara and Mundt, 2020), while correlation analysis between functional traits and environmental factors was carried out with the *pheatmap* package (Kolde, 2019). The interpretation of PCA results for functional traits was guided by a scree plot. Elbow analysis and Gap statistics were employed to determine the optimal number of clusters. Functional correlation analysis was conducted with the *corrplot* package (Taiyun and Viliam, 2024). A relational network was visualized using the *igraph* package, retaining edges with correlations above 0.5 (Csardi and Nepusz, 2006). A random forest model was used to evaluate the importance of environmental factors in shaping functional traits. The *randomForest* (Liaw and Wiener, 2002) and *rfPermute* (Archer, 2023) packages were utilized for model construction and prediction. Unlike the standard randomForest function, the rfPermute function was chosen for its permutation-based approach to variable importance, yielding more robust results. The model was configured with 500 trees and 1,000 bootstrap iterations to enhance predictive accuracy. Model confidence and significance were tested using the *A3* package (Fortmann-Roe, 2015), yielding p-values of < 0.05, which confirmed the reliability of the predictions. Map data utilized in this study were sourced from the Alibaba Cloud data visualization platform.

## 3 Result

### 3.1 Descriptive statistics of functional traits

In LN, the distribution of most materials was concentrated in the range of 25-50 and 50-75; in LA, the LA values of most materials were distributed between 12-15 and 15-18; in LL, the range of 8-12 contains the vast majority of materials; in LW, the range of 2.5-3.5 is the most concentrated; the distribution peak of LSF appears at 1.8. In the L:W ratio, the distribution of most materials was concentrated in the range of 2.5-3.0 (**Supplementary Figure S1**). The height distribution of most materials was in the range of 4.0-6.0 and 6.0-8.0; the range of 20-40 contains the most materials; the SN value of most materials was 8; the SNL values were distributed in the range of 0-10, covering the vast majority of materials (**Supplementary Figure S2**). Additionally, the CV of functional traits of *T. mongolicus* varied significantly across different regions, indicating that thyme exhibits considerable differentiation in its functional traits in response to regional variations (**Supplementary Table S2**).

### 3.2 Cluster analysis of the functional traits of *T. mongolicus*

**Figure 1:**
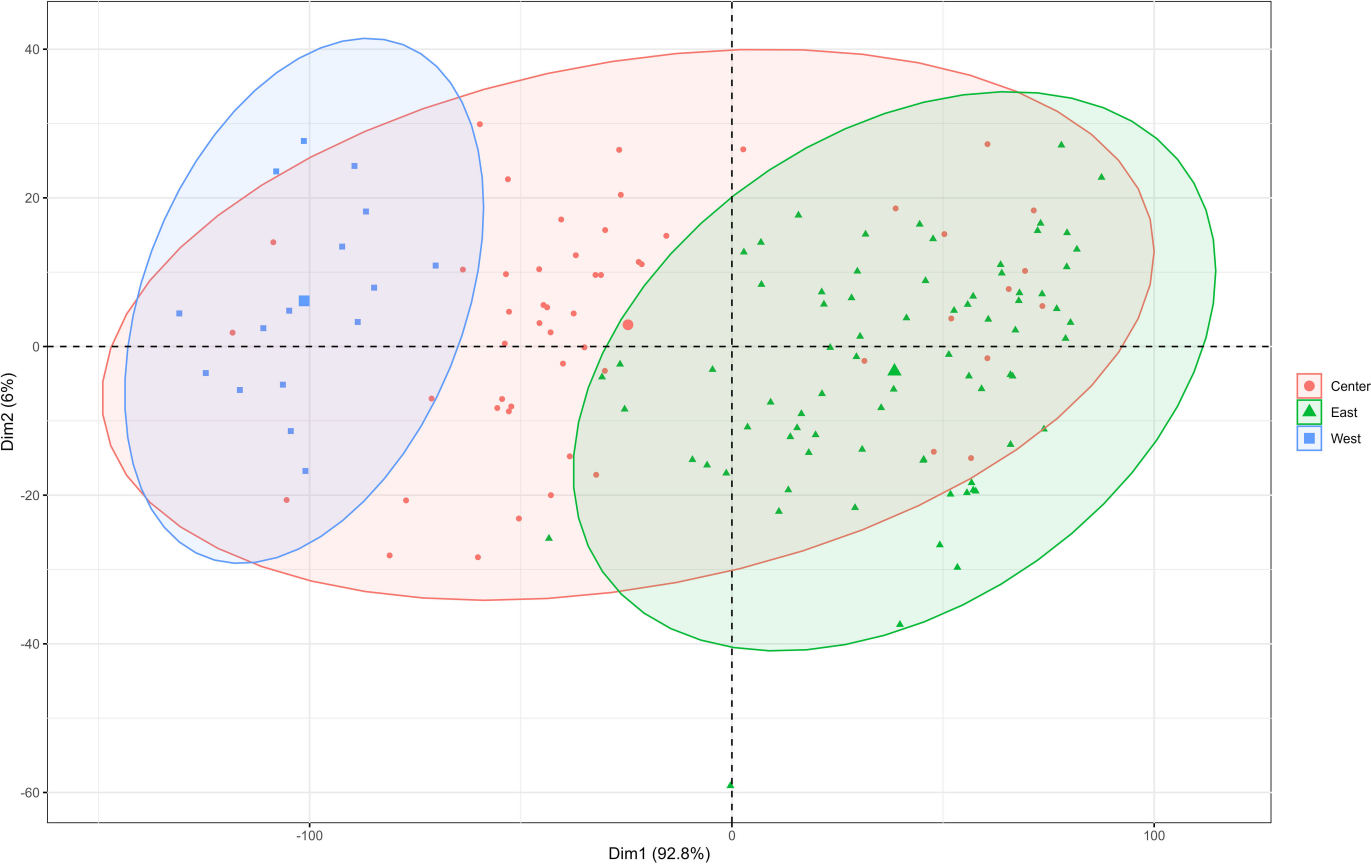
PCA was performed on the functional traits of *T. mongolicus* according to the sampling points.

This study utilized PCA to examine the functional traits of *T. mongolicus* from two perspectives. First, PCA was conducted based on the spatial distribution of sampling points. The first two principal components of the gravel map explained 98.8% of the total variance, indicating a strong interpretative capacity for functional traits (**Supplementary Figure S3**). The results revealed a clear differentiation of functional traits between the eastern and western regions, with central region traits bridging the two without clear separation (**Figure 1**).

**Figure 2:**
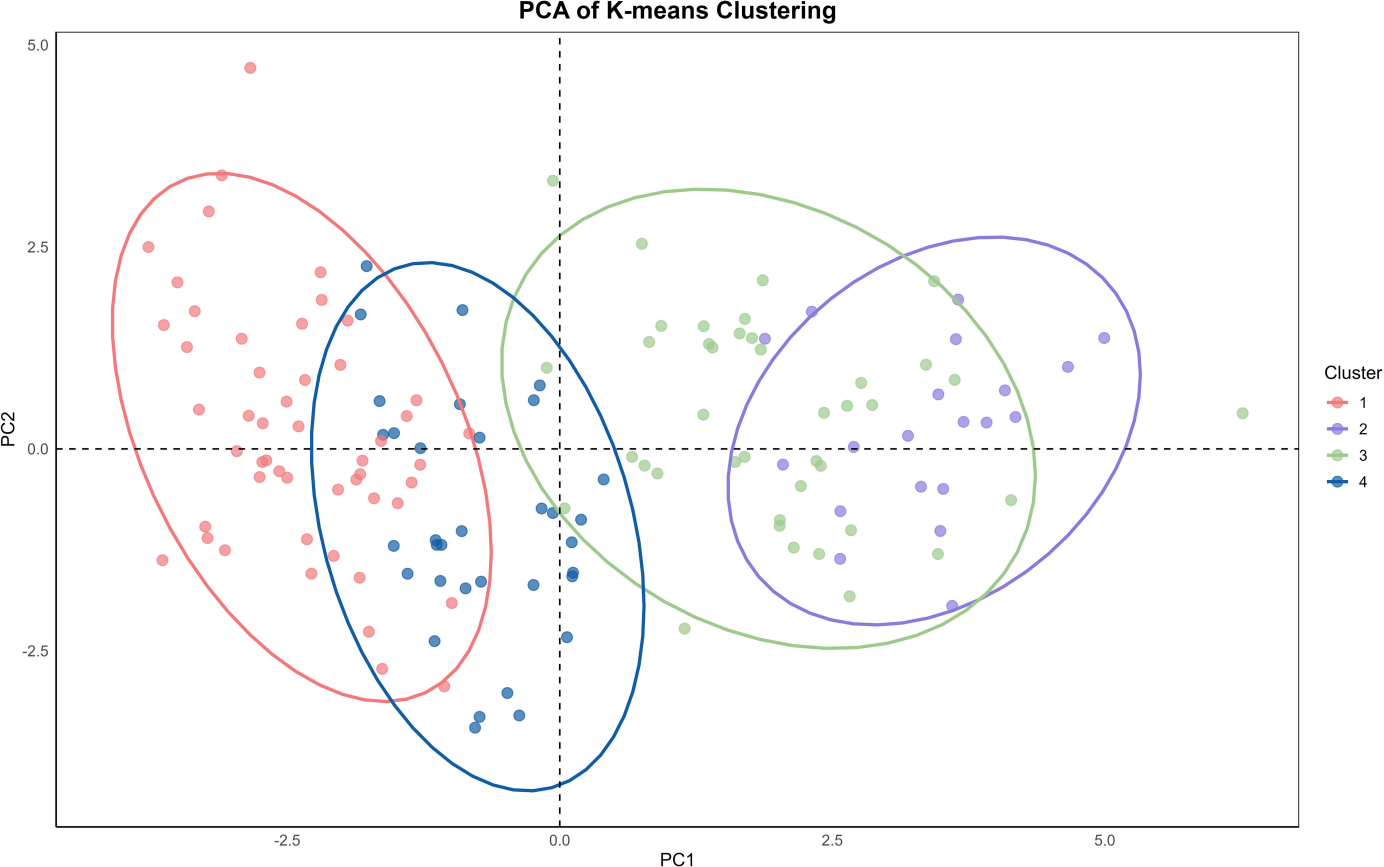
Geographical distribution of clusters based on the optimal classification results.

Subsequently, PCA was performed on the functional traits to determine the optimal clustering, identified as four clusters using elbow and GAP analyses (**Supplementary Figure S4**). The results indicated that the 145 materials could be grouped into four clusters based on their functional traits. The PCA results showed significant overlap between Clusters 1 and 4, as well as between Clusters 2 and 3. This suggests that Cluster 1 and Cluster 4 share more similarities, while Cluster 2 and Cluster 3 are more similar to each other. However, there is no overlap between Cluster 1 and Cluster 5, indicating a significant difference between them (**Figure 2**; **Supplementary Figure S5**). Based on the principal component loadings for each axis, it can be seen that LA, LW, Height, SL, and SNL have higher loadings on PC1, while LL, LSF, and L:W have higher loadings on PC2. The loading of LL is the highest on both axes, indicating that LL plays a crucial role in the functional trait variation of *T. mongolicus*.

### 3.3 Correlation analysis of the functional traits of *T. mongolicus*

**Figure 3:**
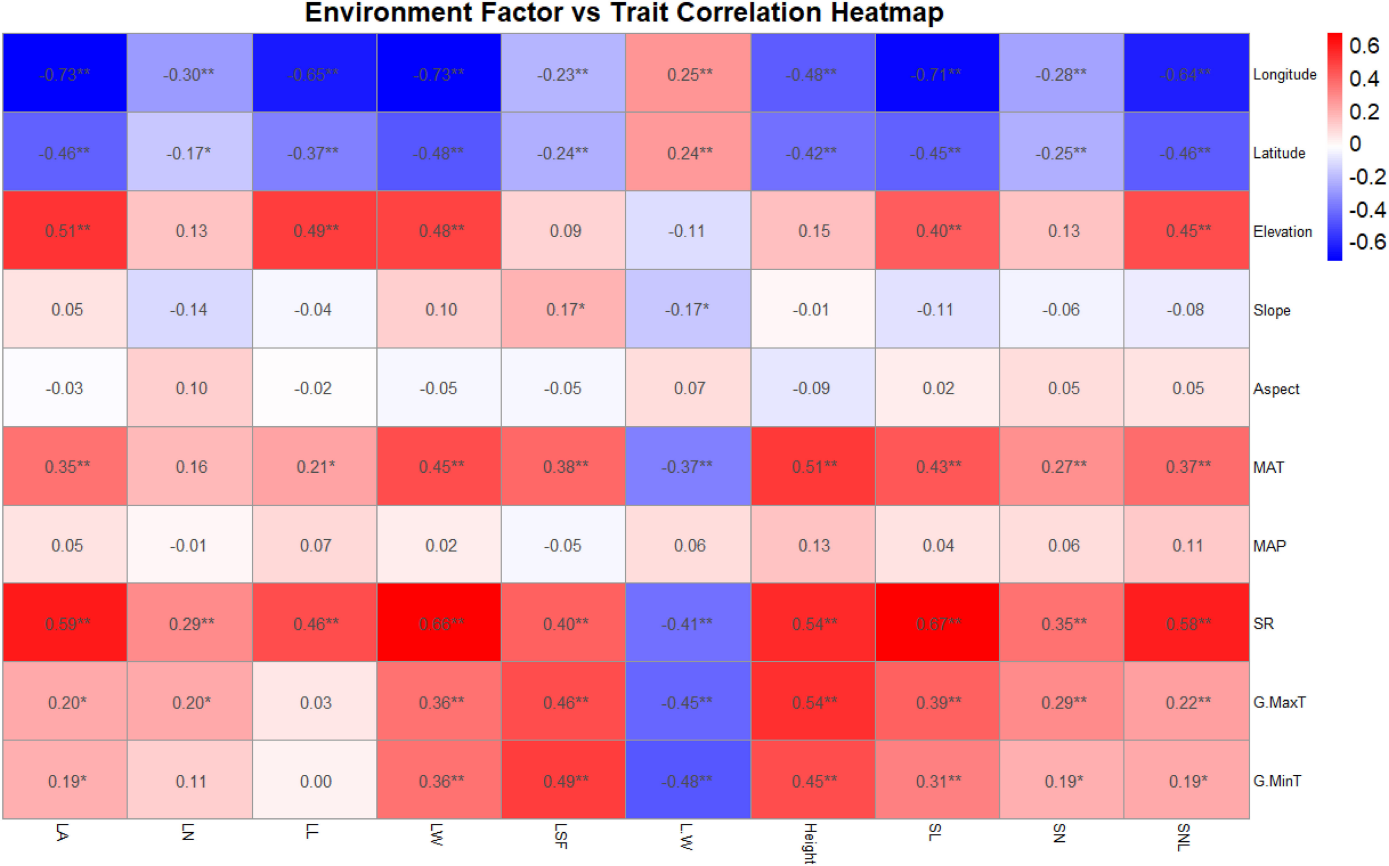
Heat map analysis of functional traits and environmental factors of *T. mongolicus*. LN, leaf number per branch; LA: leaf area; LL, leaf length; LW, leaf width; LSF, leaf shape factor; L:W, leaf length:leaf width; SL, stem length; SN, stem node number; SNL, stem node length; MAT, mean annual temperature; MAP, mean annual precipitation; SR, solar radiation; G.MaxT, growing maximum temperature; G.MinT, growing minimum temperature. The number in the figure represents the correlation coefficient, *P < 0.05; **P < 0.01.

Further analysis explored the correlations between the functional traits and environmental factors of *T. mongolicus*. Longitude and latitude exhibited significant positive correlations with the length-to-width (L:W) ratio and significant negative correlations with other functional traits. Slope, mean annual temperature (MAT), and maximum and minimum temperatures also showed significant negative correlations with L:W. Other functional shape indicators correlated negatively with latitude and longitude but showed significant positive correlations with geographic and climatic factors to varying degrees. Altitude, MAT, and GR had particularly strong effects on functional traits such as LA, LL, LW, and plant height, whereas slope and aspect showed no significant correlations (**Figure 3**).

**Figure 4:**
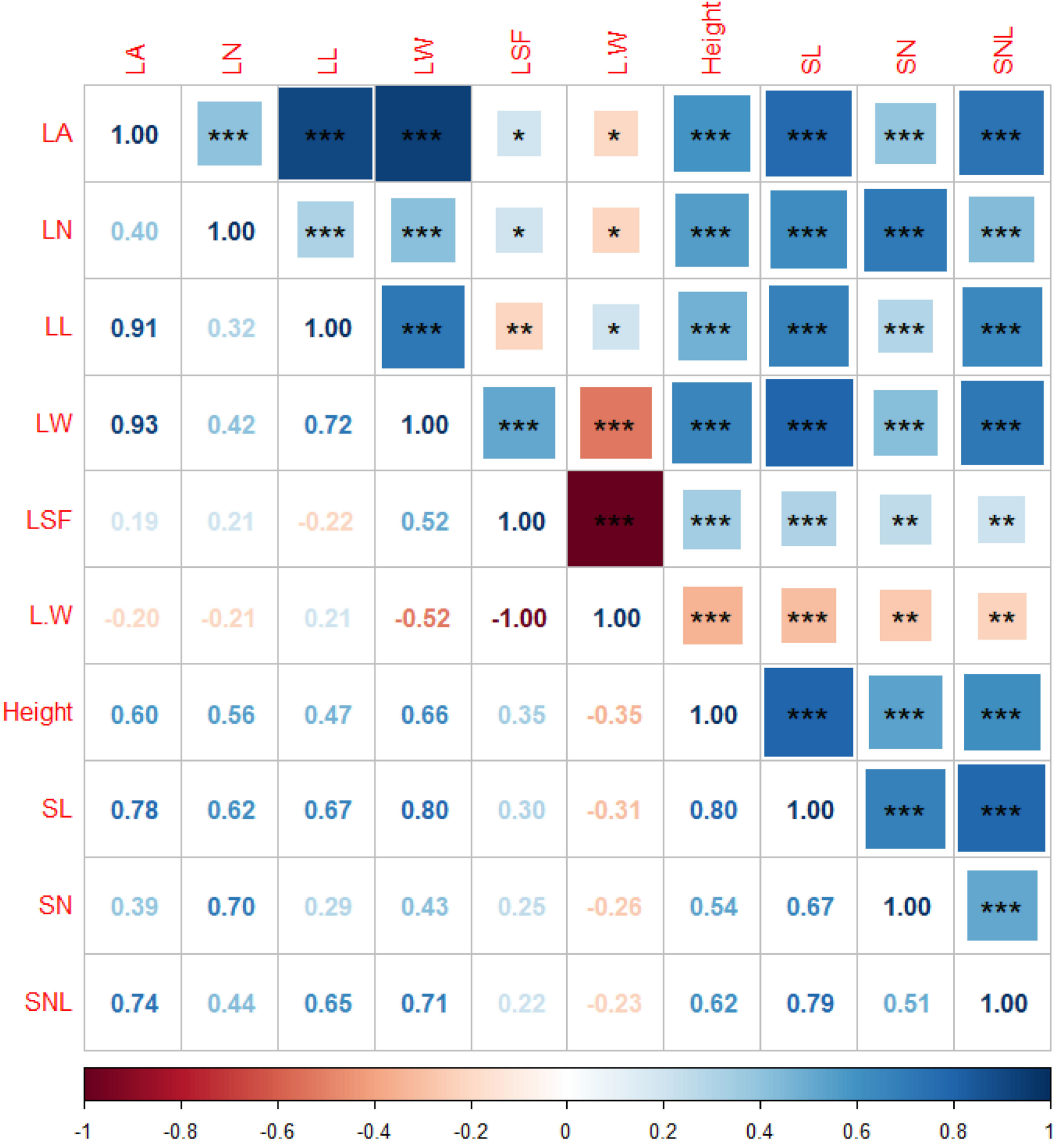
Autocorrelation analysis of the functional traits of *T. mongolicus*. LN, leaf number per branch; LA, leaf area; LL, leaf length; LW, leaf width; LSF, leaf shape factor; L:W, leaf length:leaf width; SL, stem length; SN, stem node number; SNL, stem node length. *P < 0.05; **P < 0.01, *** P < 0.001.

Finally, autocorrelation analysis highlighted strong interrelationships among the functional traits of *T. mongolicus*. LA exhibited significant positive correlations with LL and LW, while plant height and SL were similarly correlated. Other indicators also showed substantial interdependence, underscoring the intrinsic linkages between functional traits of *T. mongolicus* (**Figure 4**). In addition to the significant correlation between functional traits, there were also significant correlations between different types of environmental factors, indicating that there were also significant synergistic changes between environmental factors (**Supplementary Figure S6**).

**Figure 5:**
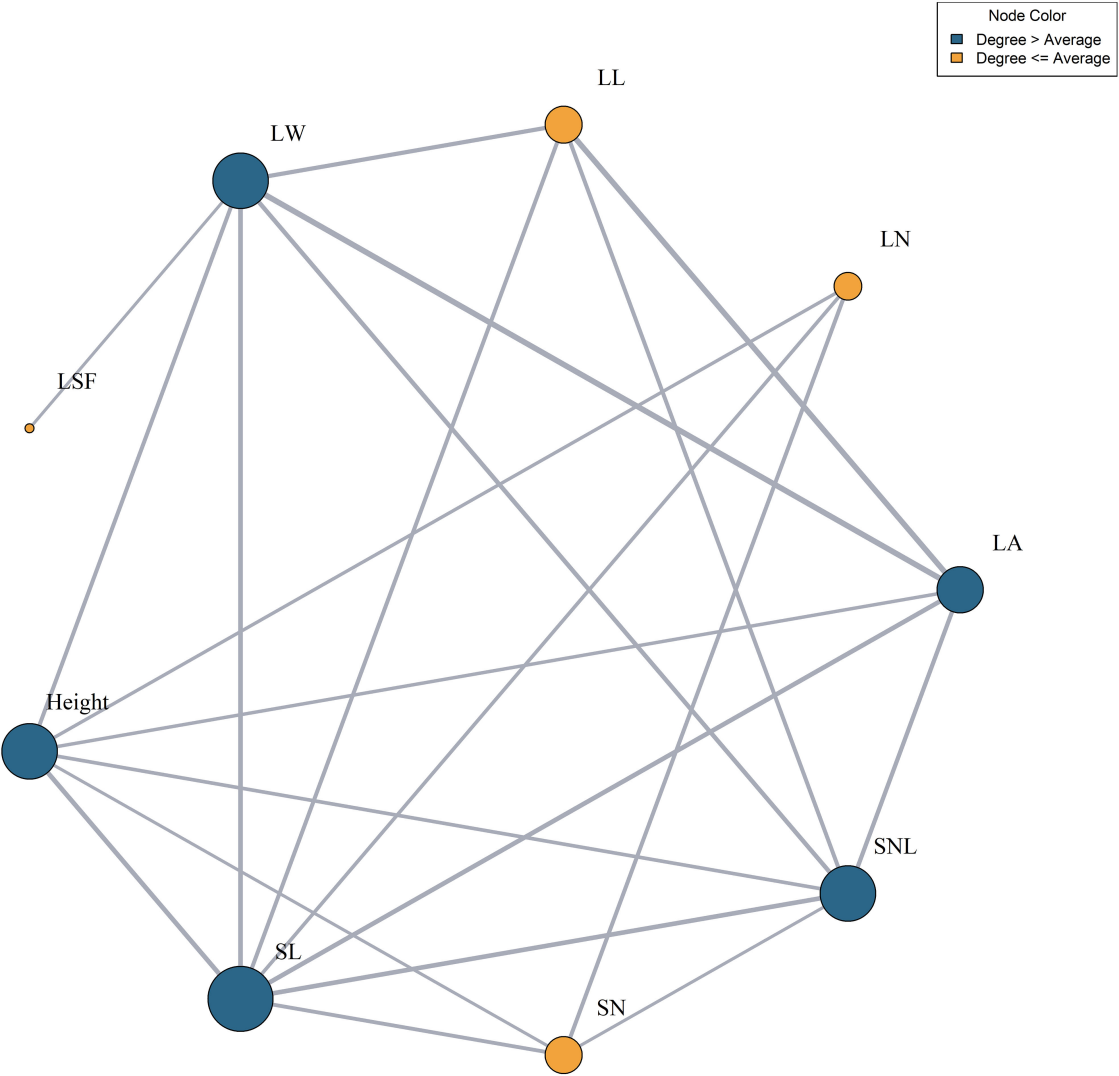
Functional trait relationship network. LN, leaf number per branch; LA, leaf area; LL, leaf length; LW, leaf width; LSF, leaf shape factor; SL, stem length; SN, stem node number; SNL, stem node length. Dark blue indicates that the connectivity is greater than the average connectivity, and yellow indicates that the connectivity is lower than the average connectivity. The larger the circle, the higher the degree of connection.

A network analysis of 10 functional trait indicators was performed, using correlations above 0.5 as the threshold for node connections. The L:W had correlations below 0.5 with all other traits and was thus excluded from the network. In the network, traits such as LW, LA, SNL, SL, and plant height had a higher number of connections than average, indicating their central roles. In contrast, traits such as LL, LN, and SN had fewer connections. SL had the highest connectivity, with seven nodes, whereas LSF had the least, with only one node (**Figure 5**).

### 3.4 Importance prediction of environmental factors

**Figure 6:**
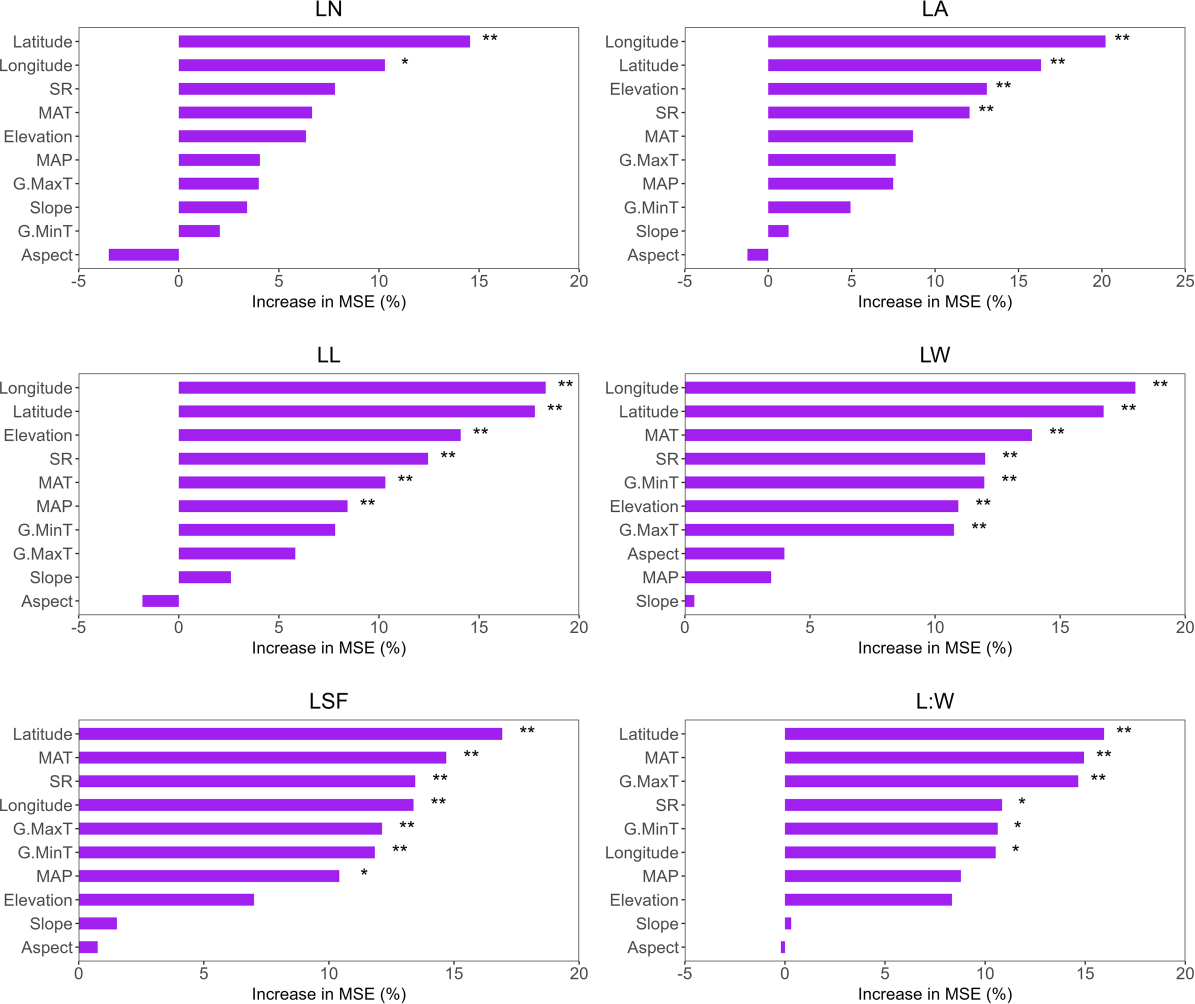
Random Forest model was used to predict the relative importance and ranking of environmental factors on the functional traits of *T. mongolicus*. MAT, mean annual temperature; MAP, mean annual precipitation; SR, solar radiation; G.MaxT, growing maximum temperature; G.MinT, growing minimum temperature. LN, leaf number per branch; LA, leaf area; LL, leaf length; LW, leaf width; LSF, leaf shape factor; L:W, leaf length:leaf width. *P < 0.05; **P < 0.01.

We used a random forest model to predict the contribution and significance of environmental factors to functional traits. In LN, latitude and longitude were the most significant factors. In LA, latitude, longitude, SR and elevation were the most significant factors. In LL, latitude, longitude, elevation, SR, MAT and mean annual precipitation (MAP) were the most significant factors. In LW, all environmental factors except aspect, MAP and slope were significant. Similarly, in LSF, all factors except elevation, slope, and aspect were significant. In L:W, all factors except MAP, elevation, slope and aspect showed notable importance (**Figure 6**).

**Figure 7:**
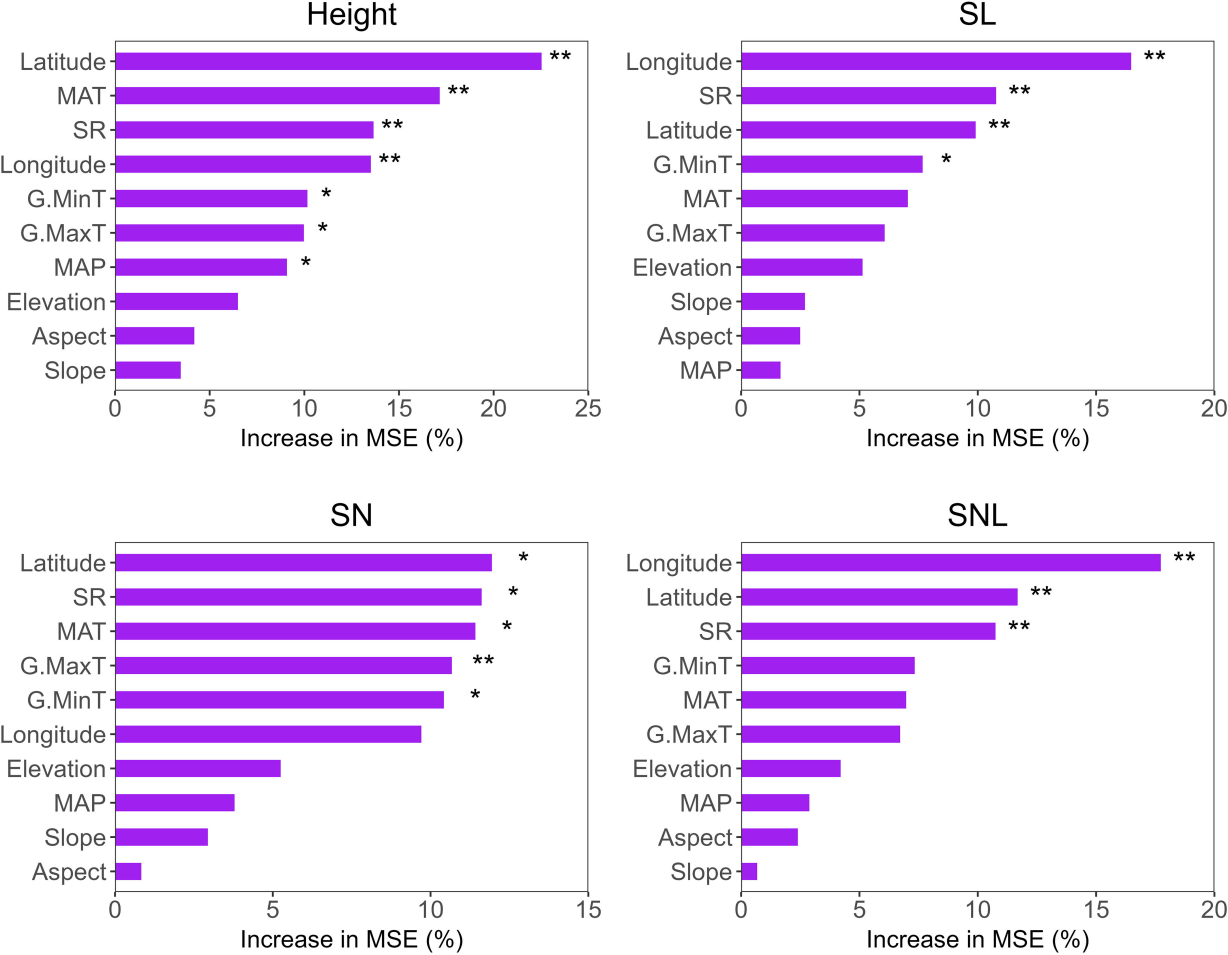
Random Forest model was used to predict the relative importance and ranking of environmental factors on the functional traits of *T. mongolicus*. MAT, mean annual temperature; MAP, mean annual precipitation; SR, solar radiation; G.MaxT, growing maximum temperature; G.MinT, growing minimum temperature. SL, stem length; SN, stem node number; SNL, stem node length. *P < 0.05; **P < 0.01.

Our results indicate that, apart from elevation, slope, and aspect, other environmental factors substantially influenced plant height. In SL, longitude, latitude, SR, and G.MinT were the primary contributors. For SN, all factors except longitude, elevation, MAP, slope, and aspect had a significant impact. For SNL, longitude, latitude, and SR showed high contributions (**Figure 7**). Overall, the random forest analysis demonstrated that latitude, longitude, and temperature-related factors were key predictors across all models.

## 4 Discussion

### 4.1 Analysis of the differential variation in the functional traits of *T. mongolicus*

Plant functional traits are shaped by a combination of genetic regulation and external environmental influences (Maugarny-Calès and Laufs, 2018). To reduce the differences in functional traits caused by different sampling times, this study adopted the method of transplanting field materials into a homogeneous garden to maintain consistency in sampling time and measurement methods to reduce external errors and improve the accuracy of the experiment. Our results revealed significant variations in the functional traits of across regions, with pronounced differences between sampling sites. This variability likely stems from two main factors. First, because thyme is a species that relies on insect pollination and has a short seed dispersal distance, limited gene flow between different regions may lead to genetic differentiation at a large scale, resulting in differences in functional traits, thus promoting the differentiation of regional characteristics (Abraham et al., 2018; Campolo et al., 2016). Second, functional traits reflect the plant’s long-term adaptation to environmental conditions (Fritz et al., 2018). The considerable environmental heterogeneity across the study area—including variations in habitat, topography, and climate—has driven significant differences in functional traits as *T. mongolicus* adapts to local conditions.

Our results demonstrated that the functional traits of *T. mongolicus* across different sites exhibited a high CV. The CV is a key metric for assessing the degree of variation in functional traits within a population and serves as an important indicator of the population’s capacity to adapt to environmental changes (Moller et al., 2023). The high CV observed in *T. mongolicus* reflects its robust adaptability to environmental variability and diversity. Furthermore, this high CV suggests that *T. mongolicus* possesses significant ecological resilience and adaptability, enabling it to cope effectively with environmental pressures. These findings align with its known biological traits of drought resistance, cold tolerance, and resilience to nutrient-poor conditions (Martin et al., 2017).

### 4.2 Relationship between functional traits and the environmental gradient

In our study, latitude and longitude showed a significant negative correlation with the remaining nine indicators, except for the L:W ratio. This is consistent with previous research, which found that functional traits exhibited significant adaptability to latitudinal and longitudinal gradients (Chang et al., 2024; Zhou et al., 2022). Our results also revealed a significant positive correlation between the functional traits of *T. mongolicus* and both MAT and solar radiation (SR). Temperature is a critical factor influencing plant growth and development as it determines photosynthetic efficiency and growth rates (Schellenberger Costa et al., 2017; Lu et al., 2016). In the Northern Hemisphere, temperature decreases significantly with increasing latitude, and plants adapt to these environmental gradients through specific functional trait combinations (Wright et al., 2004). For example, as latitude increases and temperatures decline, lower accumulated temperatures and shorter growing seasons inhibit plant growth. In response to colder climates, *T. mongolicus* adjusts its traits to minimize heat loss and enhance photosynthetic capacity. For instance, in our study, with increasing latitude, LW narrowed and LA decreased. Slender leaves helped minimize heat loss (Guerin et al., 2012). Additionally, plant height decreases to reduce heat dissipation, a response to both shorter growth cycles and lower accumulated temperatures (Moles et al., 2009). This results in a general decline in functional traits at higher latitudes compared to lower latitudes. Similarly, in northern China, where temperature decreases with increasing longitude, the functional trait trends along the longitude gradient mirror those along the latitude gradient. Additionally, a significant positive correlation was observed between LA and elevation. This may be due to the increase in altitude, resulting in more direct and strong sunlight. In such an environment, plants may increase the surface area involved in photosynthesis by increasing LA and may absorb more sunlight to ensure sufficient energy supply (Sharma et al., 2024; Wei et al., 2021).

The L:W reflects the plant’s environmental adaptation strategy. The slender leaf shape is more effective at retaining heat and water compared to the wider leaf shape (Schellenberger Costa et al., 2017). Our study found that the L:W ratio of *T. mongolicus* increased significantly with longitude, and the leaf shape was slender. Indicating an evolutionary trend toward reducing water loss and conserving heat in response to colder and drier environments (Fan et al., 2020). Conversely, L:W exhibited a significant negative correlation with temperature and rainfall. In warmer and wetter environments, plants adapt by dissipating heat more efficiently and improving water use efficiency (Toscano et al., 2019; Yavas et al., 2024). At lower latitudes, broader leaves help maintain temperature and water balance, thereby enhancing photosynthetic capacity.

### 4.3 Synergistic correlation among functional traits

Plants function as a continuum, with various organs interconnected to perform distinct functions, cooperate, influence each other, and collectively regulate life activities. To adapt to diverse environments, plant organs interact dynamically, forming an “optimal collocation” (Guittar et al., 2016; He et al., 2020). Our research identified SL as the trait with the highest number of nodes and the strongest associations with other functional traits. This prominence arises because the stem serves as the primary conduit for nutrient transport and a structural base for leaf growth (Zhang et al., 2020). These attributes directly determine the number of leaves, nutrient availability during leaf development, and the integration of *T. mongolicus* leaves with other organs. Consequently, SL exhibits more nodes compared to other functional traits.

Among leaf attributes, LW and LA have demonstrated higher-than-average node counts. This is because LW and LA are highly plastic traits that frequently change in response to environmental conditions, enhancing adaptation. Changes in these traits significantly influence photosynthetic capacity and nutrient acquisition, which, in turn, impact other functional traits of *T. mongolicus* (Gong et al., 2020; Zhang et al., 2019). The observed strong correlations between LW, LA, and environmental factors—especially MAT—further support this conclusion.

## 5 Conclusion

Our findings revealed that *T. mongolicus* exhibited differentiation in functional traits to adapt to varied environments, leading to significant trait variation across different locations. In this adaptive process, the temperature gradient associated with latitude and longitude was a key factor influencing functional traits. Furthermore, multiple traits interacted closely during adaptation, with SL, LW, and LA playing critical roles. These results elucidated the variation pattern of *T. mongolicus* in northern China and identified key factors driving functional trait variation. Such insights established a solid foundation for future breeding efforts targeting *T. mongolicus*.

## Data Availability

The original contributions presented in the study are included in the article/**Supplementary Material**. Further inquiries can be directed to the corresponding author.
