# Supplementary material for: Changes in the functional traits of Thymus mongolicus along environmental gradients and factors influencing the traits of Northern China
Source: Front Plant Sci. 2025 May 20;16:1596849. doi: 10.3389/fpls.2025.1596849 (PMC12130018; doi:10.3389/fpls.2025.1596849)
Supplement: Supplementary file 1 [file DataSheet1.docx]

Supplementary Material


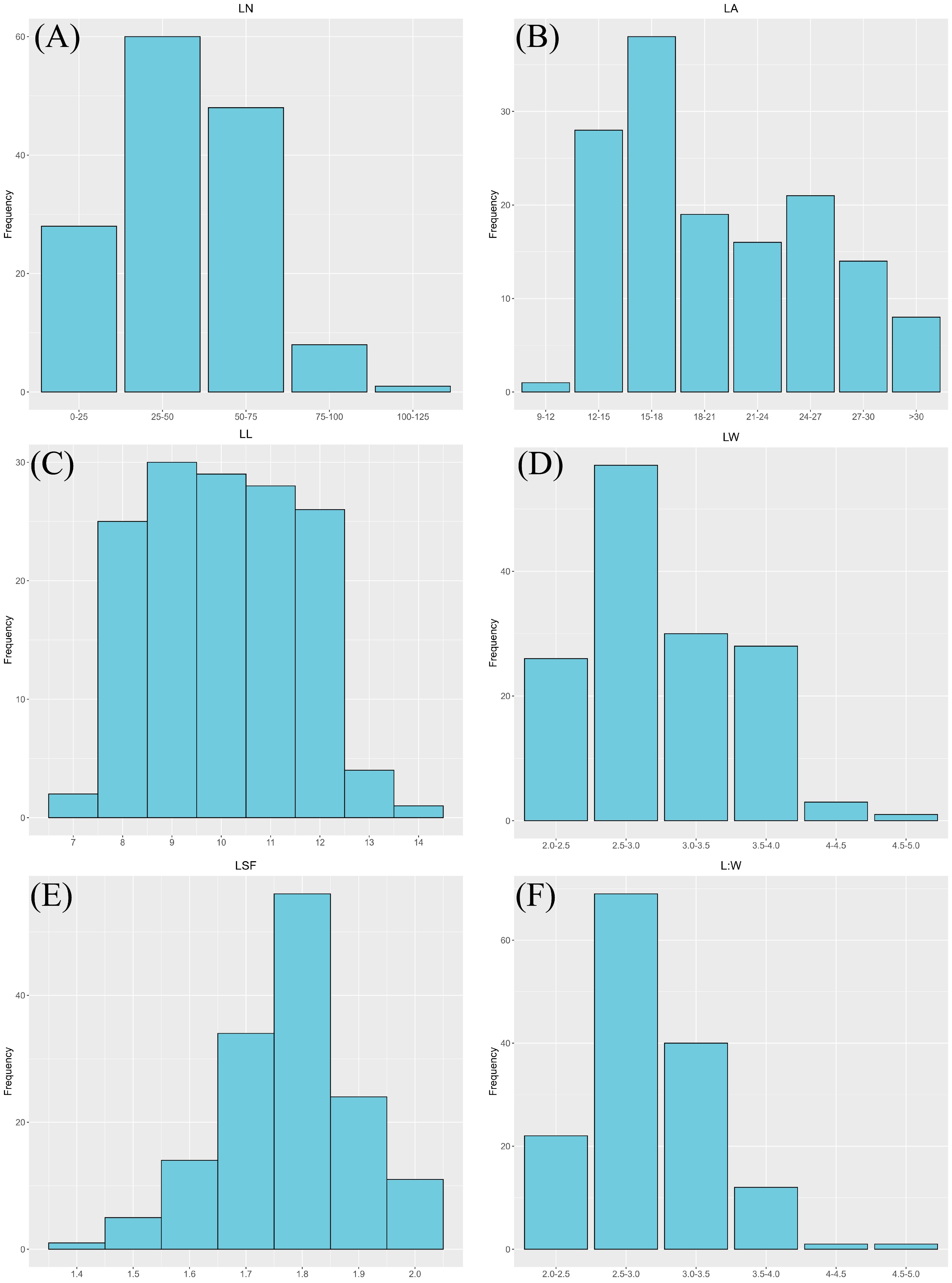


**Figure S1** Leaf trait frequency distribution map. LN, leaf number per branch; LA: leaf area; LL, leaf length; LW, leaf width; LSF, leaf shape factor; L:W, leaf length: leaf width


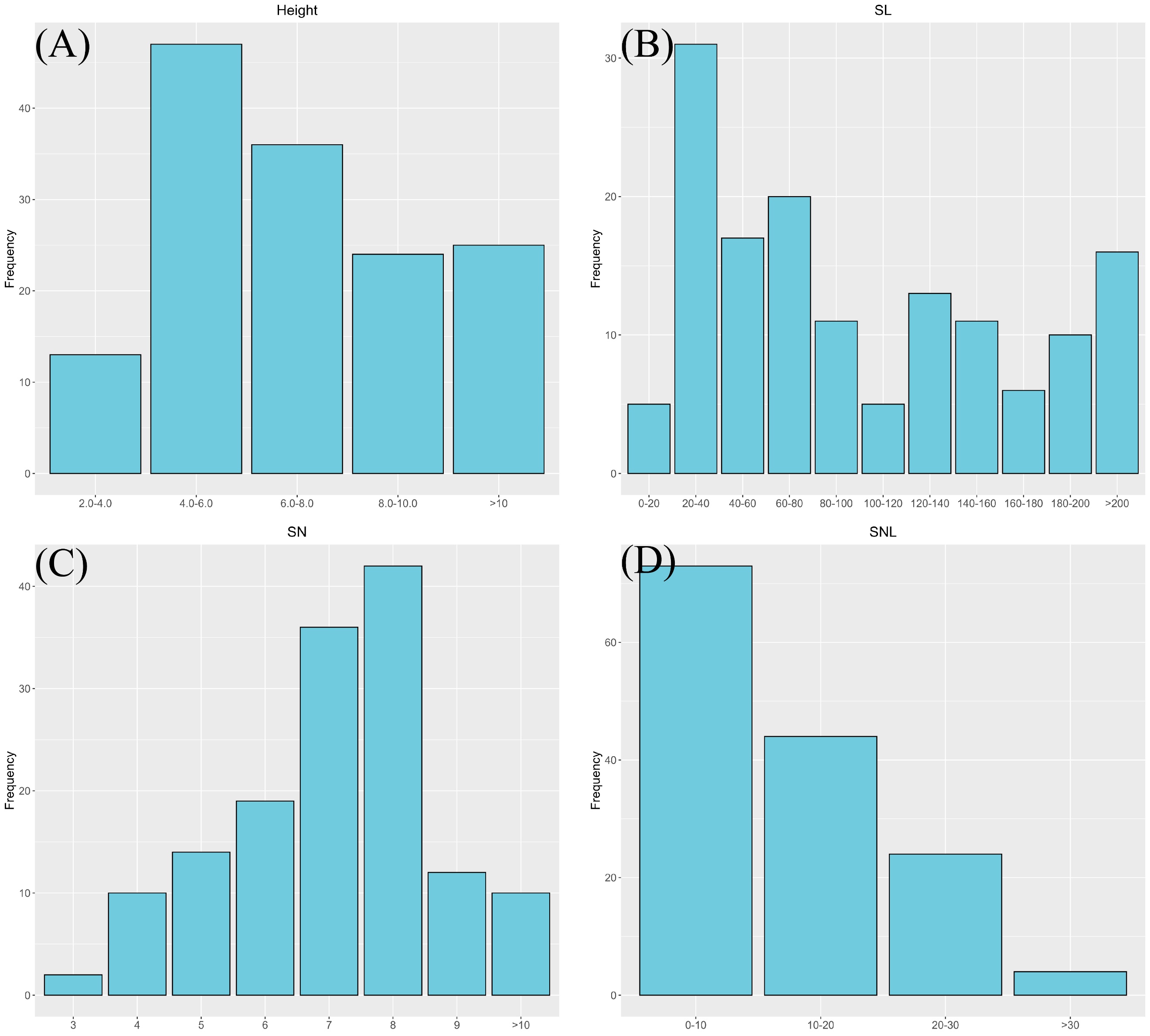


**Figure S2** Stem trait frequency distribution map. SL, stem length; SN, stem node number; SNL, stem node length;


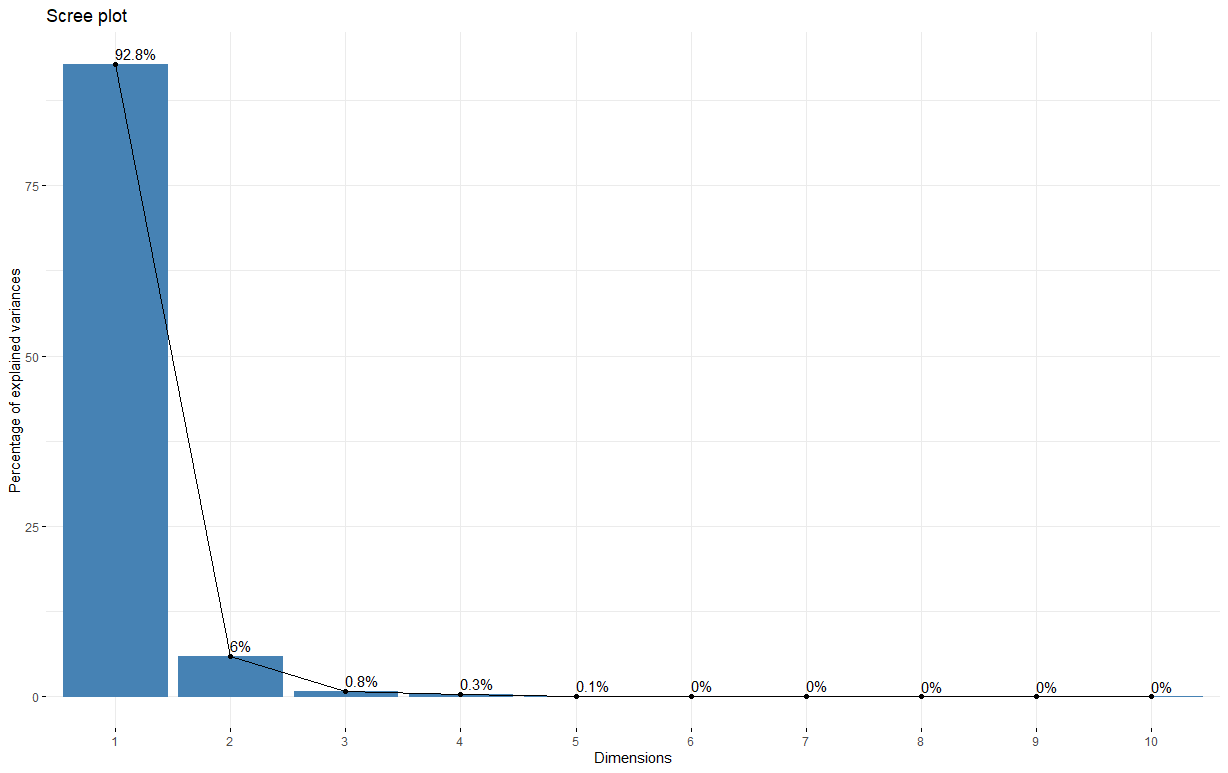


**Figure S3** PCA analysis of gravel map.


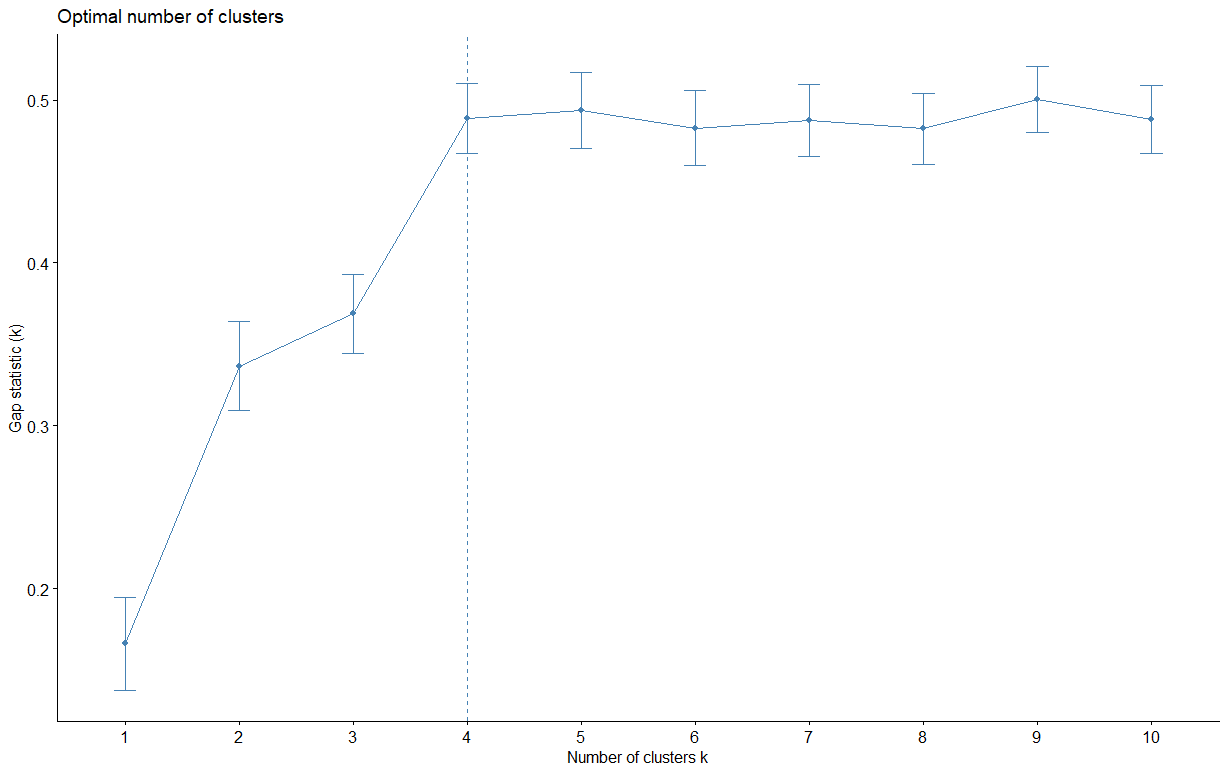


**Figure S4** Gap analysis determines the optimal number of clusters. The dotted line represents the optimal number of clusters.


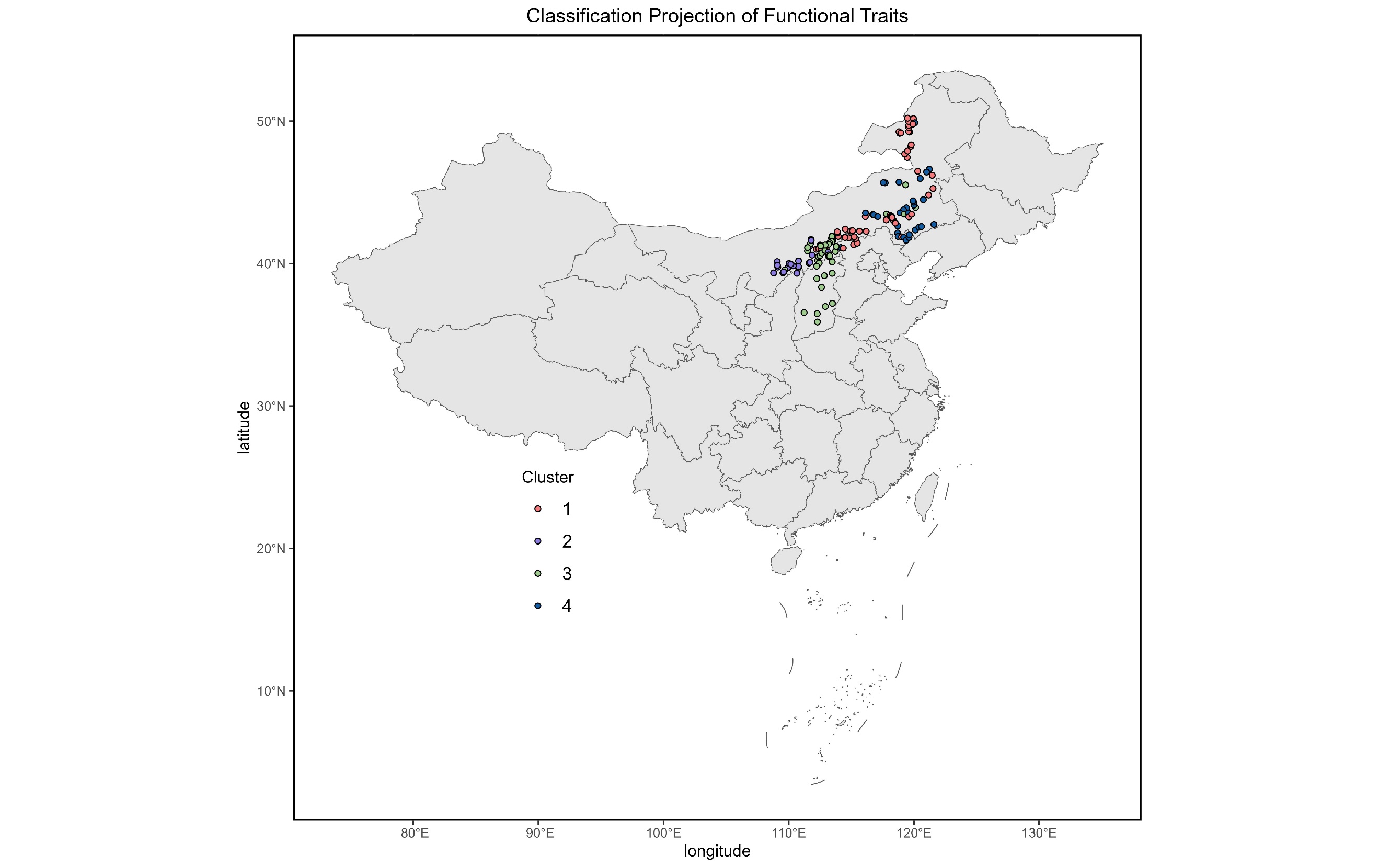


**Figure S5** Based on the spatial distribution of the various clusters in the optimal classification.


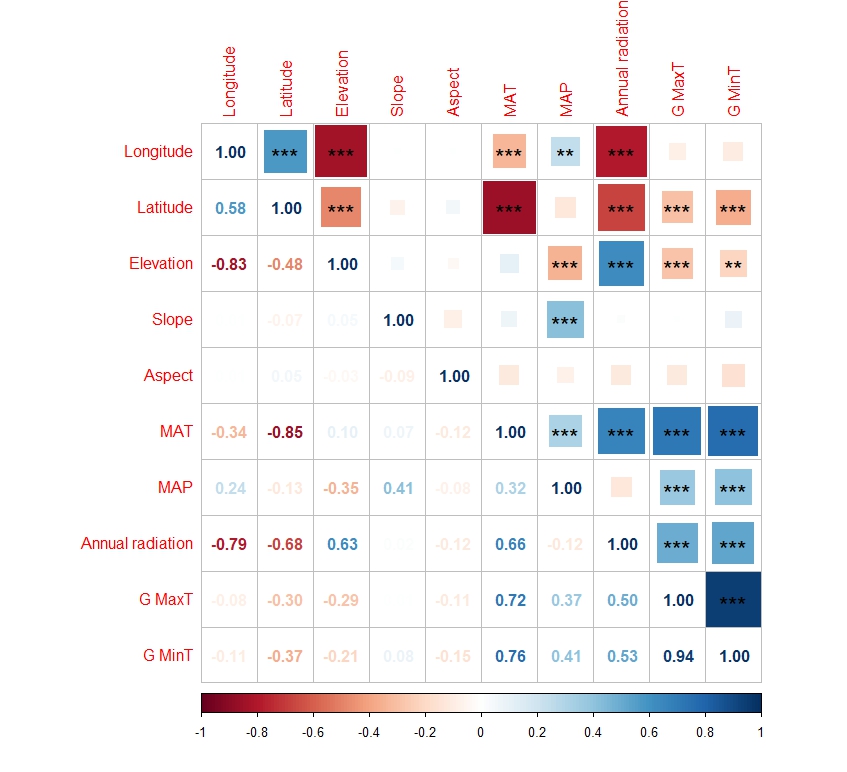


**Figure S6** Autocorrelation analysis of environment factors. MAT, mean annual temperature; MAP, mean annual precipitation; SR, solar radiation; G.MaxT, growing maximum temperature; G.MinT, growing minimum temperature.

| Area | MAT | MAP | Elevation | Slope | Aspect | Solar radiation | G.MaxT | G.MinT |
| --- | --- | --- | --- | --- | --- | --- | --- | --- |
| West | 3.17±0.71 b | 375.19±12.43 a | 984.21±109.28 b | 1.74±0.24 a | 174.02±27.38 a | 181177.25±2349.20 b | 19.85±0.45 a | 6.56±0.47 a |
| Center | 3.46±0.41 b | 357.63±11.82 a | 1105.03±58.37 a | 1.42±0.16 b | 162.01±12.31 a | 184369.32±1269.82 ab | 19.93±0.26 a | 6.54±0.30 a |
| East | 4.58±0.30 a | 258.43±20.03 b | 1093.44±33.69 ab | 1.35±0.12 b | 172.16±11.58a | 187677.32±999.93 a | 20.83±0.20 a | 7.14± 0.22 a |

**Table S1** Comparison of environmental factors across different regions.

Note: Different lowercase letters indicate statistically significant differences between groups. P<0.05. G.MaxT, growing maximum temperature; G.MinT, growing minimum temperature.

**Table S3** Loadings of each principal component on the principal axes PC1 and PC2.

|  | PC1 | PC2 |
| --- | --- | --- |
| LN | 0.28 | -0.03 |
| LA | 0.36 | 0.24 |
| LL | 0.28 | 0.47 |
| LW | 0.38 | 0.01 |
| LSF | 0.19 | -0.56 |
| L:W | -0.19 | 0.56 |
| Height | 0.34 | -0.02 |
| SL | 0.39 | 0.08 |
| SN | 0.29 | -0.08 |
| SNL | 0.34 | 0.14 |

Note: LN, leaf number per branch; LA, leaf area; LL, leaf length; LW, leaf width; LSF, leaf shape factor; SL, stem length; SN, stem node number; SNL, stem node length
